# *trans*-(1,8-Dibenzyl-1,3,6,8,10,13-hexa­azacyclo­tetra­deca­ne)diisonicotinatonickel(II)

**DOI:** 10.1107/S1600536808001116

**Published:** 2008-01-16

**Authors:** Jeong Hyeong Han, Bong Gon Kim, Kil Sik Min

**Affiliations:** a Department of Chemistry Education, Kyungpook National University, Daegu 702-701, Republic of Korea; b Department of Chemistry Education and Research Institute of Natural Science, Gyeongsang National University, Chinju 660-701, Republic of Korea

## Abstract

In the centrosymmetric title compound, [Ni(C_6_H_4_NO_2_)_2_(C_22_H_34_N_6_)], the Ni^II^ ion is bonded to the four secondary N atoms of the macrocyclic ligand in a square-planar fashion and two carboxyl­ate O atoms of the isonicotinate ions in axial positions, resulting in a tetra­gonally distorted octa­hedron. An offset face-to-face π–π stacking inter­action [centroid–centroid distance = 3.674(4) Å] and N—H⋯N and N—H⋯O hydrogen-bonding inter­actions give rise to a one-dimensional supra­molecular structure in the solid state.

## Related literature

For related literature, see Hancock (1990 ▶); Jung *et al.* (1989 ▶); Larionova *et al.* (2003 ▶); Lee & Suh (2004 ▶); Shetty *et al.* (1996 ▶); Tsuge *et al.* (2004 ▶).
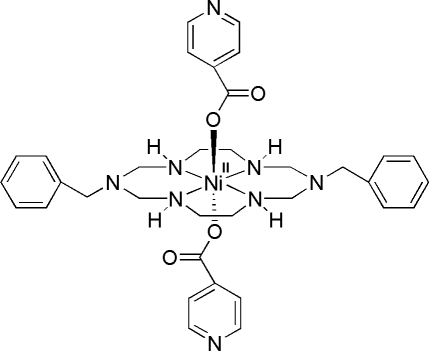


## Experimental

### 

#### Crystal data

[Ni(C_6_H_4_NO_2_)_2_(C_22_H_34_N_6_)]
*M* *_r_* = 685.45
Monoclinic, 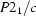

*a* = 8.3418 (5) Å
*b* = 17.3104 (9) Å
*c* = 10.9596 (6) Å
β = 91.892 (1)°
*V* = 1581.70 (15) Å^3^
*Z* = 2
Mo *K*α radiation
μ = 0.67 mm^−1^
*T* = 173 (2) K
0.40 × 0.20 × 0.20 mm

#### Data collection

Siemens SMART CCD diffractometer
Absorption correction: multi-scan (*SADABS*; Sheldrick, 1996 ▶) *T*_min_ = 0.778, *T*_max_ = 0.875
9843 measured reflections
3671 independent reflections
3288 reflections with *I* > 2σ(*I*)
*R*_int_ = 0.022

#### Refinement

*R*[*F* ^2^ > 2σ(*F* ^2^)] = 0.062
*wR*(*F* ^2^) = 0.116
*S* = 1.29
3671 reflections
214 parameters
H-atom parameters constrained
Δρ_max_ = 0.52 e Å^−3^
Δρ_min_ = −0.56 e Å^−3^

### 

Data collection: *SMART* (Siemens, 1996 ▶); cell refinement: *SAINT* (Siemens, 1996 ▶); data reduction: *SHELXTL* (Sheldrick, 2008 ▶); program(s) used to solve structure: *SHELXS97* (Sheldrick, 2008 ▶); program(s) used to refine structure: *SHELXL97* (Sheldrick, 2008 ▶); molecular graphics: *ORTEP-3* (Farrugia, 1997 ▶); software used to prepare material for publication: *SHELXL97*.

## Supplementary Material

Crystal structure: contains datablocks global, I. DOI: 10.1107/S1600536808001116/pk2081sup1.cif

Structure factors: contains datablocks I. DOI: 10.1107/S1600536808001116/pk2081Isup2.hkl

Additional supplementary materials:  crystallographic information; 3D view; checkCIF report

## Figures and Tables

**Table 1:** Hydrogen-bond geometry (Å, °)

*D*—H⋯*A*	*D*—H	H⋯*A*	*D*⋯*A*	*D*—H⋯*A*
N1—H1⋯O2	0.93	1.97	2.838 (3)	154
N2—H2⋯N4^i^	0.93	2.31	3.160 (3)	152

Symmetry code: (i) 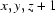.

**Fig. 1.**
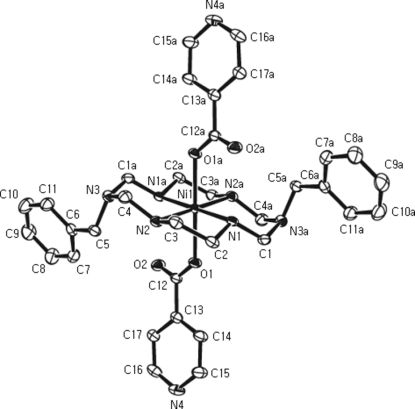
View of the molecular structure of the title compound showing 50% displacement ellipsoids for the non-hydrogen atoms. Atoms labeled with the suffix a are at the symmetry position (1 - x, 1 - y, 2 - z).

**Fig. 2.**
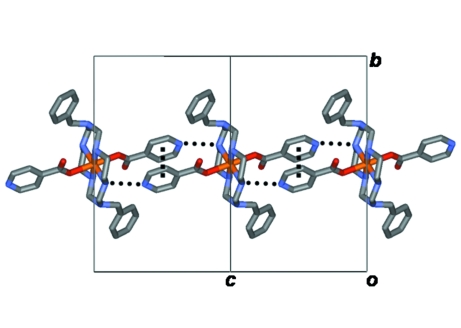
Perspective view of the title compound showing a 1-D chain along the c axis by hydrogen bonding interactions (black circles) and offset face-to-face π-π interactions (black squares).

## supplementary crystallographic information

### Comment

The coordination chemistry of tetraaza macrocyclic ligands has been extensively studied in the context of metalloenzymes and the construction of extended supramolecular networks (Tsuge *et al.*, 2004; Larionova *et al.*, 2003). In particular, Ni^II^ macrocyclic complexes with vacant axial positions are good candidates for assembling novel multi-dimensional materials in which they can possess interesting properties (Lee & Suh, 2004). Here, we report the synthesis and structure of the title compound.

As shown in Fig. 1, the Ni^II^ ion is coordinated by the four secondary amine N atoms of the macrocyclic ligand in a square-planar fashion and two oxygen atoms from isonicotinate ions at the axial positions, resulting in a tetragonally distorted octahedron. The average Ni—N and Ni—O bond distances are 2.062 (1) and 2.159 (1) Å, respectively. The axial Ni—O bond distance is longer than the equatorial Fe—N bond lengths, which can be attributed to the Jahn-Teller distortion of the Ni^II^ ion and/or the ring contraction of the macrocyclic ligand. Two CO bond distances of the carboxylate group are not significantly different although one is coordinated (1.246 (4) Å) and the other is uncoordinated (1.266 (3) Å) to the Ni^II^ ion. The complex has an inversion center at the Ni atom and the azamacrocyclic ligand adopts thermodynamically the most stable *R,R,S,S* configuration (Hancock, 1990). The geometry of the tertiary nitrogen atom N3 is normal; C—N distances average 1.458 (2) Å and C—N—C angles are in the range 111.8 (2)–115.7 (2)°, which is indicative of significant contribution of *sp*^2^ hybridization for the nitrogen atom. The shortest Ni···Ni intrachain separation within the one-dimensional chain is 10.960 (1) Å and is 31% greater than the shortest interchain Ni···Ni distance of 8.342 (1) Å.

All pyridine groups of the isonicotinates coordinating Ni^II^ ions axially are involved in offset face-to-face π-π stacking interactions (centroid···centroid 3.674 (4) Å), which leads to a supramolecular one-dimensional polymer propagating along the *c* axis (Fig. 2). The pyridine rings are positioned completely parallel to each other (dihedral angle of 0.0°). The interplanar separation and the offset angle between the ring planes of isonicotinate ions are 3.545 (4) Å and 9.71 (9)°, respectively (Shetty *et al.*, 1996).

Within a one-dimensional chain, the non-coordinated carbonyl oxygen atom of the carboxylate ion forms an intramolecular hydrogen bond with the secondary amine (N1) of the macrocycle. In addition, the nitrogen atom of isonicotinate ion forms an intermolecular hydrogen bond with the secondary amine (N2) of the macrocycle which joins the molecules into a robust one-dimensional polymer (Table 1).

### Experimental

The starting complex, [Ni(C_22_H_34_N_6_)Cl_2_], used in this work was prepared by a literature procedure (Jung *et al.*, 1989). To a DMF/H_2_O (*v*/*v*; 1:1, 20 ml) solution of [Ni(C_22_H_34_N_6_)Cl_2_] (0.20 g, 0.40 mmol) was added dropwise an MeCN solution (10 ml) containing isonicotinic acid (0.10 g, 0.80 mmol) and excess triethylamine (0.08 g, 0.80 mmol) at room temperature. The color of the solution turned from yellow to pale pink. The mixture solution was stirred for 1 h during which time a pink precipitate of formed which was collected by filtration, washed with MeCN, and dried in air. Single crystals of the title compound suitable for X-ray crystallography were obtained by layering of the MeCN solution of isonicotinate on the DMF/H_2_O solution of [Ni(C_22_H_34_N_6_)Cl_2_] for several days. Yield 0.21 g (77%).

### Refinement

All H atoms in the title compound were placed in geometrically idealized positions and constrained to ride on their parent atoms, with C—H distances of 0.95 (ring H atoms) or 0.99 (open chain H atoms) Å and N—H distance of 0.93 Å, and with *U*_iso_(H) values of 1.2 times the equivalent anisotropic displacement parameters of the parent C and N atoms.

### Crystal data

**Table:**

[Ni(C_6_H_4_NO_2_)_2_(C_22_H_34_N_6_)]	*F*_000_ = 724
*M**_r_* = 685.45	*D*_x_ = 1.439 Mg m^−^^3^
Monoclinic, *P*2_1_/*c*	Mo *K*α radiation λ = 0.71073 Å
Hall symbol: -P 2ybc	Cell parameters from 5240 reflections
*a* = 8.3418 (5) Å	θ = 2.2–28.1º
*b* = 17.3104 (9) Å	µ = 0.67 mm^−^^1^
*c* = 10.9596 (6) Å	*T* = 173 (2) K
β = 91.892 (1)º	Block, pink
*V* = 1581.70 (15) Å^3^	0.40 × 0.20 × 0.20 mm
*Z* = 2	

### Data collection

**Table:**

Siemens SMART CCD diffractometer	3671 independent reflections
Radiation source: fine-focus sealed tube	3288 reflections with *I* > 2σ(*I*)
Monochromator: graphite	*R*_int_ = 0.022
*T* = 173(2) K	θ_max_ = 28.3º
φ and ω scans	θ_min_ = 2.4º
Absorption correction: multi-scan(SADABS; Sheldrick, 1996)	*h* = −10→9
*T*_min_ = 0.778, *T*_max_ = 0.875	*k* = −22→19
9843 measured reflections	*l* = −14→13

### Refinement

**Table:**

Refinement on *F*^2^	Secondary atom site location: difference Fourier map
Least-squares matrix: full	Hydrogen site location: inferred from neighbouring sites
*R*[*F*^2^ > 2σ(*F*^2^)] = 0.062	H-atom parameters constrained
*wR*(*F*^2^) = 0.116	*w* = 1/[σ^2^(*F*_o_^2^) + (0.0189*P*)^2^ + 2.8532*P*] where *P* = (*F*_o_^2^ + 2*F*_c_^2^)/3
*S* = 1.29	(Δ/σ)_max_ < 0.001
3671 reflections	Δρ_max_ = 0.52 e Å^−^^3^
214 parameters	Δρ_min_ = −0.55 e Å^−^^3^
Primary atom site location: structure-invariant direct methods	Extinction correction: none

### Special details

**Table:**

Geometry. All e.s.d.'s (except the e.s.d. in the dihedral angle between two l.s. planes) are estimated using the full covariance matrix. The cell e.s.d.'s are taken into account individually in the estimation of e.s.d.'s in distances, angles and torsion angles; correlations between e.s.d.'s in cell parameters are only used when they are defined by crystal symmetry. An approximate (isotropic) treatment of cell e.s.d.'s is used for estimating e.s.d.'s involving l.s. planes.
Refinement. Refinement of *F*^2^ against ALL reflections. The weighted *R*-factor *wR* and goodness of fit *S* are based on *F*^2^, conventional *R*-factors *R* are based on *F*, with *F* set to zero for negative *F*^2^. The threshold expression of *F*^2^ > 2σ(*F*^2^) is used only for calculating *R*-factors(gt) *etc*. and is not relevant to the choice of reflections for refinement. *R*-factors based on *F*^2^ are statistically about twice as large as those based on *F*, and *R*- factors based on ALL data will be even larger.

### Fractional atomic coordinates and isotropic or equivalent isotropic displacement parameters (Å^2^)

**Table:**

	*x*	*y*	*z*	*U*_iso_*/*U*_eq_	
Ni1	0.5000	0.5000	1.0000	0.01570 (13)	
O1	0.4295 (2)	0.54216 (12)	0.82075 (17)	0.0223 (4)	
O2	0.1901 (3)	0.48759 (12)	0.77448 (18)	0.0279 (5)	
N1	0.2943 (3)	0.43532 (13)	1.0093 (2)	0.0185 (5)	
H1	0.2407	0.4385	0.9337	0.022*	
N2	0.3733 (3)	0.58652 (13)	1.0846 (2)	0.0195 (5)	
H2	0.4031	0.5862	1.1671	0.023*	
N3	0.5746 (3)	0.68651 (14)	1.0464 (2)	0.0216 (5)	
N4	0.3459 (3)	0.59376 (16)	0.3712 (2)	0.0295 (6)	
C1	0.3199 (4)	0.35247 (17)	1.0367 (3)	0.0231 (6)	
H1A	0.3657	0.3476	1.1209	0.028*	
H1B	0.2147	0.3260	1.0340	0.028*	
C2	0.1956 (3)	0.47603 (17)	1.0988 (2)	0.0216 (6)	
H2A	0.2370	0.4653	1.1828	0.026*	
H2B	0.0832	0.4579	1.0915	0.026*	
C3	0.2034 (3)	0.56208 (17)	1.0729 (3)	0.0228 (6)	
H3A	0.1603	0.5730	0.9895	0.027*	
H3B	0.1385	0.5908	1.1318	0.027*	
C4	0.4065 (3)	0.66490 (17)	1.0359 (3)	0.0239 (6)	
H4A	0.3711	0.6669	0.9488	0.029*	
H4B	0.3425	0.7032	1.0805	0.029*	
C5	0.6325 (3)	0.68978 (17)	1.1745 (2)	0.0226 (6)	
H5A	0.6392	0.6365	1.2069	0.027*	
H5B	0.5527	0.7181	1.2223	0.027*	
C6	0.7938 (3)	0.72810 (16)	1.1938 (3)	0.0220 (6)	
C7	0.9015 (4)	0.69899 (18)	1.2817 (3)	0.0299 (7)	
H7	0.8761	0.6531	1.3244	0.036*	
C8	1.0459 (4)	0.7364 (2)	1.3077 (3)	0.0361 (8)	
H8	1.1184	0.7161	1.3681	0.043*	
C9	1.0843 (4)	0.8026 (2)	1.2465 (3)	0.0367 (8)	
H9	1.1830	0.8282	1.2645	0.044*	
C10	0.9780 (4)	0.8320 (2)	1.1580 (3)	0.0386 (8)	
H10	1.0045	0.8777	1.1150	0.046*	
C11	0.8342 (4)	0.79516 (18)	1.1324 (3)	0.0291 (7)	
H11	0.7620	0.8159	1.0720	0.035*	
C12	0.3141 (3)	0.52339 (16)	0.7488 (2)	0.0200 (6)	
C13	0.3282 (3)	0.54825 (16)	0.6159 (2)	0.0206 (6)	
C14	0.4444 (4)	0.59945 (18)	0.5799 (3)	0.0268 (6)	
H14	0.5202	0.6200	0.6378	0.032*	
C15	0.4485 (4)	0.6204 (2)	0.4576 (3)	0.0300 (7)	
H15	0.5290	0.6558	0.4340	0.036*	
C16	0.2331 (4)	0.54476 (19)	0.4082 (3)	0.0280 (7)	
H16	0.1572	0.5258	0.3490	0.034*	
C17	0.2206 (4)	0.52022 (17)	0.5277 (3)	0.0231 (6)	
H17	0.1394	0.4846	0.5489	0.028*	

### Atomic displacement parameters (Å^2^)

**Table:**

	*U*^11^	*U*^22^	*U*^33^	*U*^12^	*U*^13^	*U*^23^
Ni1	0.0150 (2)	0.0204 (2)	0.0117 (2)	−0.0036 (2)	−0.00054 (16)	0.00087 (19)
O1	0.0235 (10)	0.0286 (11)	0.0145 (9)	−0.0042 (8)	−0.0035 (7)	0.0012 (8)
O2	0.0286 (11)	0.0330 (12)	0.0216 (10)	−0.0085 (9)	−0.0051 (8)	0.0058 (9)
N1	0.0199 (11)	0.0240 (12)	0.0116 (10)	−0.0038 (9)	−0.0006 (9)	−0.0001 (9)
N2	0.0185 (11)	0.0245 (12)	0.0153 (11)	−0.0030 (9)	−0.0017 (9)	0.0005 (9)
N3	0.0226 (12)	0.0229 (12)	0.0190 (12)	−0.0041 (10)	−0.0021 (9)	−0.0005 (9)
N4	0.0334 (14)	0.0398 (16)	0.0154 (12)	0.0069 (12)	0.0004 (10)	0.0028 (11)
C1	0.0277 (15)	0.0266 (15)	0.0150 (13)	−0.0063 (12)	0.0015 (11)	0.0025 (11)
C2	0.0186 (13)	0.0308 (15)	0.0155 (13)	−0.0044 (11)	0.0008 (10)	−0.0033 (11)
C3	0.0185 (14)	0.0298 (16)	0.0201 (14)	−0.0013 (12)	0.0003 (11)	−0.0023 (11)
C4	0.0223 (14)	0.0260 (15)	0.0233 (15)	0.0005 (12)	−0.0027 (11)	0.0022 (12)
C5	0.0256 (15)	0.0257 (15)	0.0166 (13)	−0.0043 (12)	0.0004 (11)	−0.0016 (11)
C6	0.0236 (15)	0.0222 (14)	0.0204 (14)	−0.0019 (11)	0.0016 (11)	−0.0065 (11)
C7	0.0361 (18)	0.0229 (15)	0.0304 (16)	−0.0040 (13)	−0.0032 (13)	0.0029 (13)
C8	0.0323 (18)	0.0322 (18)	0.043 (2)	−0.0009 (14)	−0.0156 (15)	0.0015 (15)
C9	0.0275 (17)	0.039 (2)	0.043 (2)	−0.0089 (15)	−0.0075 (14)	−0.0031 (16)
C10	0.0380 (19)	0.0323 (18)	0.045 (2)	−0.0127 (15)	−0.0030 (16)	0.0091 (15)
C11	0.0291 (16)	0.0286 (16)	0.0292 (16)	−0.0034 (13)	−0.0052 (13)	0.0047 (13)
C12	0.0248 (14)	0.0204 (13)	0.0146 (12)	0.0026 (11)	−0.0015 (10)	−0.0002 (10)
C13	0.0223 (14)	0.0217 (14)	0.0178 (13)	0.0070 (11)	−0.0007 (10)	−0.0019 (11)
C14	0.0297 (16)	0.0338 (17)	0.0166 (14)	−0.0012 (13)	−0.0032 (11)	−0.0001 (12)
C15	0.0321 (17)	0.0389 (18)	0.0192 (14)	−0.0034 (14)	0.0008 (12)	0.0033 (13)
C16	0.0267 (16)	0.0387 (18)	0.0184 (14)	0.0055 (13)	−0.0046 (12)	−0.0060 (12)
C17	0.0236 (14)	0.0253 (15)	0.0204 (14)	0.0018 (11)	−0.0002 (11)	−0.0019 (11)

### Geometric parameters (Å, °)

**Table:**

Ni1—N1^i^	2.054 (2)	C3—H3B	0.9900
Ni1—N1	2.054 (2)	C4—H4A	0.9900
Ni1—N2^i^	2.070 (2)	C4—H4B	0.9900
Ni1—N2	2.070 (2)	C5—C6	1.509 (4)
Ni1—O1^i^	2.1591 (19)	C5—H5A	0.9900
Ni1—O1	2.1591 (19)	C5—H5B	0.9900
O1—C12	1.266 (3)	C6—C11	1.389 (4)
O2—C12	1.246 (4)	C6—C7	1.390 (4)
N1—C2	1.479 (3)	C7—C8	1.389 (5)
N1—C1	1.479 (4)	C7—H7	0.9500
N1—H1	0.9300	C8—C9	1.371 (5)
N2—C3	1.480 (3)	C8—H8	0.9500
N2—C4	1.487 (4)	C9—C10	1.388 (5)
N2—H2	0.9300	C9—H9	0.9500
N3—C4	1.452 (4)	C10—C11	1.380 (4)
N3—C1^i^	1.453 (4)	C10—H10	0.9500
N3—C5	1.470 (4)	C11—H11	0.9500
N4—C15	1.337 (4)	C12—C13	1.527 (4)
N4—C16	1.340 (4)	C13—C14	1.380 (4)
C1—N3^i^	1.453 (4)	C13—C17	1.386 (4)
C1—H1A	0.9900	C14—C15	1.391 (4)
C1—H1B	0.9900	C14—H14	0.9500
C2—C3	1.518 (4)	C15—H15	0.9500
C2—H2A	0.9900	C16—C17	1.384 (4)
C2—H2B	0.9900	C16—H16	0.9500
C3—H3A	0.9900	C17—H17	0.9500
			
N1^i^—Ni1—N1	180.00 (11)	H3A—C3—H3B	108.4
N1^i^—Ni1—N2^i^	86.12 (9)	N3—C4—N2	113.4 (2)
N1—Ni1—N2^i^	93.88 (9)	N3—C4—H4A	108.9
N1^i^—Ni1—N2	93.88 (9)	N2—C4—H4A	108.9
N1—Ni1—N2	86.12 (9)	N3—C4—H4B	108.9
N2^i^—Ni1—N2	180.000 (1)	N2—C4—H4B	108.9
N1^i^—Ni1—O1^i^	91.53 (8)	H4A—C4—H4B	107.7
N1—Ni1—O1^i^	88.47 (8)	N3—C5—C6	114.5 (2)
N2^i^—Ni1—O1^i^	92.00 (8)	N3—C5—H5A	108.6
N2—Ni1—O1^i^	88.00 (8)	C6—C5—H5A	108.6
N1^i^—Ni1—O1	88.47 (8)	N3—C5—H5B	108.6
N1—Ni1—O1	91.53 (8)	C6—C5—H5B	108.6
N2^i^—Ni1—O1	88.00 (8)	H5A—C5—H5B	107.6
N2—Ni1—O1	92.00 (8)	C11—C6—C7	118.5 (3)
O1^i^—Ni1—O1	180.000 (1)	C11—C6—C5	121.9 (3)
C12—O1—Ni1	131.18 (18)	C7—C6—C5	119.4 (3)
C2—N1—C1	114.0 (2)	C8—C7—C6	120.6 (3)
C2—N1—Ni1	104.91 (16)	C8—C7—H7	119.7
C1—N1—Ni1	115.03 (18)	C6—C7—H7	119.7
C2—N1—H1	107.5	C9—C8—C7	120.3 (3)
C1—N1—H1	107.5	C9—C8—H8	119.8
Ni1—N1—H1	107.5	C7—C8—H8	119.8
C3—N2—C4	114.8 (2)	C8—C9—C10	119.6 (3)
C3—N2—Ni1	104.84 (17)	C8—C9—H9	120.2
C4—N2—Ni1	113.30 (17)	C10—C9—H9	120.2
C3—N2—H2	107.9	C11—C10—C9	120.3 (3)
C4—N2—H2	107.9	C11—C10—H10	119.9
Ni1—N2—H2	107.9	C9—C10—H10	119.9
C4—N3—C1^i^	115.7 (2)	C10—C11—C6	120.7 (3)
C4—N3—C5	111.8 (2)	C10—C11—H11	119.6
C1^i^—N3—C5	115.5 (2)	C6—C11—H11	119.6
C15—N4—C16	116.4 (3)	O2—C12—O1	127.4 (3)
N3^i^—C1—N1	114.2 (2)	O2—C12—C13	116.5 (2)
N3^i^—C1—H1A	108.7	O1—C12—C13	116.1 (2)
N1—C1—H1A	108.7	C14—C13—C17	118.0 (3)
N3^i^—C1—H1B	108.7	C14—C13—C12	122.1 (3)
N1—C1—H1B	108.7	C17—C13—C12	119.9 (3)
H1A—C1—H1B	107.6	C13—C14—C15	118.9 (3)
N1—C2—C3	108.4 (2)	C13—C14—H14	120.5
N1—C2—H2A	110.0	C15—C14—H14	120.5
C3—C2—H2A	110.0	N4—C15—C14	123.8 (3)
N1—C2—H2B	110.0	N4—C15—H15	118.1
C3—C2—H2B	110.0	C14—C15—H15	118.1
H2A—C2—H2B	108.4	N4—C16—C17	123.8 (3)
N2—C3—C2	108.1 (2)	N4—C16—H16	118.1
N2—C3—H3A	110.1	C17—C16—H16	118.1
C2—C3—H3A	110.1	C16—C17—C13	119.1 (3)
N2—C3—H3B	110.1	C16—C17—H17	120.4
C2—C3—H3B	110.1	C13—C17—H17	120.4
			
N1^i^—Ni1—O1—C12	172.2 (2)	C3—N2—C4—N3	−178.8 (2)
N1—Ni1—O1—C12	−7.8 (2)	Ni1—N2—C4—N3	−58.4 (3)
N2^i^—Ni1—O1—C12	86.0 (2)	C4—N3—C5—C6	−167.4 (2)
N2—Ni1—O1—C12	−94.0 (2)	C1^i^—N3—C5—C6	57.5 (3)
N2^i^—Ni1—N1—C2	164.18 (17)	N3—C5—C6—C11	42.2 (4)
N2—Ni1—N1—C2	−15.82 (17)	N3—C5—C6—C7	−142.4 (3)
O1^i^—Ni1—N1—C2	72.27 (17)	C11—C6—C7—C8	0.3 (5)
O1—Ni1—N1—C2	−107.73 (17)	C5—C6—C7—C8	−175.3 (3)
N2^i^—Ni1—N1—C1	38.05 (18)	C6—C7—C8—C9	−0.2 (5)
N2—Ni1—N1—C1	−141.95 (18)	C7—C8—C9—C10	−0.2 (6)
O1^i^—Ni1—N1—C1	−53.85 (18)	C8—C9—C10—C11	0.4 (6)
O1—Ni1—N1—C1	126.15 (18)	C9—C10—C11—C6	−0.4 (5)
N1^i^—Ni1—N2—C3	165.31 (17)	C7—C6—C11—C10	0.0 (5)
N1—Ni1—N2—C3	−14.69 (17)	C5—C6—C11—C10	175.4 (3)
O1^i^—Ni1—N2—C3	−103.29 (17)	Ni1—O1—C12—O2	17.8 (4)
O1—Ni1—N2—C3	76.71 (17)	Ni1—O1—C12—C13	−162.89 (18)
N1^i^—Ni1—N2—C4	39.44 (19)	O2—C12—C13—C14	168.4 (3)
N1—Ni1—N2—C4	−140.56 (19)	O1—C12—C13—C14	−11.0 (4)
O1^i^—Ni1—N2—C4	130.83 (18)	O2—C12—C13—C17	−10.5 (4)
O1—Ni1—N2—C4	−49.17 (18)	O1—C12—C13—C17	170.1 (3)
C2—N1—C1—N3^i^	−176.2 (2)	C17—C13—C14—C15	−0.1 (4)
Ni1—N1—C1—N3^i^	−54.9 (3)	C12—C13—C14—C15	−179.0 (3)
C1—N1—C2—C3	170.1 (2)	C16—N4—C15—C14	0.7 (5)
Ni1—N1—C2—C3	43.4 (2)	C13—C14—C15—N4	−0.1 (5)
C4—N2—C3—C2	167.2 (2)	C15—N4—C16—C17	−1.3 (5)
Ni1—N2—C3—C2	42.2 (2)	N4—C16—C17—C13	1.1 (5)
N1—C2—C3—N2	−59.6 (3)	C14—C13—C17—C16	−0.4 (4)
C1^i^—N3—C4—N2	72.6 (3)	C12—C13—C17—C16	178.6 (3)
C5—N3—C4—N2	−62.3 (3)		

Symmetry codes: (i) −*x*+1, −*y*+1, −*z*+2.

### Hydrogen-bond geometry (Å, °)

**Table:**

*D*—H···*A*	*D*—H	H···*A*	*D*···*A*	*D*—H···*A*
N1—H1···O2	0.93	1.97	2.838 (3)	154
N2—H2···N4^ii^	0.93	2.31	3.160 (3)	152

Symmetry codes:  (ii) *x*, *y*, *z*+1.
